# Individual contributions of climate and vegetation change to soil moisture trends across multiple spatial scales

**DOI:** 10.1038/srep32782

**Published:** 2016-09-07

**Authors:** Huihui Feng

**Affiliations:** 1 Key Laboratory of Watershed Geographic Sciences, Nanjing Institute of Geography and Limnology, Chinese Academy of Sciences, Nanjing, 21008, China

## Abstract

Climate and vegetation change are two dominating factors for soil moisture trend. However, their individual contributions remain unknown due to their complex interaction. Here, I separated their contributions through a trajectory-based method across the global, regional and local scales. Our results demonstrated that climate change accounted for 98.78% and 114.64% of the global drying and wetting trend. Vegetation change exhibited a relatively weak influence (contributing 1.22% and −14.64% of the global drying and wetting) because it occurred in a limited area on land. Regionally, the impact of vegetation change cannot be neglected, which contributed −40.21% of the soil moisture change in the wetting zone. Locally, the contributions strongly correlated to the local environmental characteristics. Vegetation negatively affected soil moisture trends in the dry and sparsely vegetated regions and positively in the wet and densely vegetated regions. I conclude that individual contributions of climate and vegetation change vary at the global, regional and local scales. Climate change dominates the soil moisture trends, while vegetation change acts as a regulator to drying or wetting the soil under the changing climate.

Soil moisture is a critical state variable for the Earth’s system, as its temporal and spatial variations strongly influence the global water and energy cycles^1^,^2^. In recent decades, many regions on land experienced significant drying or wetting trends, which triggered serious social, ecological and environment problems^3^,^4^,^5^,^6^,^7^,^8^. Identification of the driving forces and their contributions will be helpful for effective management of water resources and climatic adaptation. Several factors could be attributed to these trends, which can be summarized as natural and anthropogenic effects^9^,^10^. The former is characterized by climate change^3^,^11^,^12^, while the latter often is represented by vegetation change^13^,^14^. Generally, climate change affects water supply and demand, while vegetation change strongly influences water distribution by altering the surface properties^10^. The driving forces interact to create spatial and temporal patterns of the terrestrial water. Numerous studies have been undertaken to capture the controlling factors of water change. Many scientists hold the views that climate dominates the water dynamics^3^,^15^, while others believe that human plays increasing impacts^13^,^16^,^17^,^18^. The controversy arises from the interdependent nature of factors and their complex interaction. Clarification of the individual contributions remains as a challenge, leaving a great gap for understanding the mechanism of global water cycle.

Hydrological models and satellite remote sensing are usually used to monitor global soil moisture. The models estimate soil moisture by physical interactions between the atmospheric and environmental factors. However, their performances are usually weakened due to the complex physical interactions in them are simplified. In recent decades, satellite remote sensing has been widely adopted to retrieve surface soil moisture for its spatially consistent view. Here, I use long-term satellite data sets and trajectory-based method to clarify the individual contributions of climate and vegetation change on the soil moisture trend. The Climate Change Initiative (CCI) soil moisture data set of the European Space Agency (ESA) is adopted to monitor the global water trend from 1982 to 2013. The normalized difference vegetation index (NDVI), observed from the Global Inventory Monitoring and Modeling System (GIMMS) NDVI3g (third generation GIMMS NDVI from AVHRR sensors), is used to capture the vegetation trend. That is because the indicator could provide a quantitatively way to evaluate the land use and cover change. Specifically, the introductions of CCI and GIMMS NDVI data sets are shown in the Methods section. Methodologically, linear regression is firstly used to examine the trends of soil moisture and vegetation change for its simple form. Then vegetation change regions (vegetation degradation, expansion and non-vegetation/change) are extracted from the land. Subsequently, soil moisture change in non-vegetation/change regions is taken as a synthetic result of climate influences and served as a reference for isolating the contributions of vegetation change in the vegetation degradation and expansion regions (see the Methods section)^19^. Considering the spatial variability of terrestrial water patterns, the contributions of vegetation change are evaluated in the drying (soil moisture trend < 0 and significance < 0.05) and wetting (trend > 0 and significance < 0.05) zones. Both zones comprise the three vegetation change regions (vegetation degradation, expansion and non-vegetation/change regions). Understanding the terrestrial water trend requires investigating its driving forces over a broad range of scales. Thus, the individual contributions of vegetation change are evaluated at the global, regional and local scales. Globally, the contributions are identified in the drying and wetting zones. Regionally, the contributions are calculated for the vegetation change regions in the drying and wetting zones. Locally, the contribution is mapped at specific sites over land.

Figure 1 shows the temporal trends of global soil moisture and vegetation change in the past 30 years, with the multi-year mean values of soil moisture and NDVI are 0.229 cm^3^/cm^3^ and 0.404, respectively. The global soil moisture decreases slightly with a change rate of −0.002 per decade (R^2^ = 0.04, p = 0.264). Of the land, 22.76% get drying and 8.76% get wetting, which is consistent with the previous studies evaluated from satellite-retrieved^8^,^20^ or modelled data^21^,^22^. Spatially, soil moisture increases in the Amazon basin, southern Africa and north-eastern Asia and decreases in northern Africa and central Asia. The global vegetation presents a significant expansion trend, which is also consistent with previous studies^23^,^24^. The expansion rate of NDVI is 0.004 per decade (R^2^ = 0.32, p < 0.001). Specifically, 7.94% and 32.01% of the land experience vegetation degradation and expansion, with the corresponding rates of −0.014 and 0.015 per decade. The highest vegetation expansion occurs in northern North America, northern South America, central Africa, and south Asia. Vegetation degrades significantly in regions of north-central North America and southern South America. The soil moisture and vegetation trends are spatially correlated for large regions. Specifically, 7.99% and 22.52% of the drying zones experienced vegetation degradation and expansion, respectively, while these values are 7.49% and 43.19% in the wetting zones, respectively.

Further statistics show that the trends of soil moisture are heterogeneous in both drying and wetting zones. For the drying zones, soil moisture decreases by −0.025 cm^3^/cm^3^ per decade. More specifically, the highest decreasing rate occurs in the vegetation expansion regions (−0.026 cm^3^/cm^3^ per decade), followed by the non-vegetation/change regions (−0.024 cm^3^/cm^3^ per decade) and finally, the degradation regions (−0.022 cm^3^/cm^3^ per decade). If the soil moisture trend in the non-change/vegetation region is a result of climate change, the trends determined by vegetation change can be isolated in the expansion and degradation regions. Specifically, −0.002 and 0.002 cm^3^/cm^3^ per decade of the trends are generated by vegetation changes in the two vegetation change regions. It indicates that the vegetation expansion would accelerate the soil moisture decreasing in the drying zones, while the degradation would mitigate the trend. The probable reason is that vegetation strongly controls evapotranspiration, and the increase of vegetation would strongly accelerate the water deficit in the drying zones^17^,^25^,^26^. In the wetting zones, the soil moisture increases at the fastest rate in the non-change/vegetation regions (0.030 cm^3^/cm^3^ per decade), followed by the degradation (0.028 cm^3^/cm^3^ per decade) and expansion (0.021 cm^3^/cm^3^ per decade) regions. Therefore, −0.002 and −0.009 cm^3^/cm^3^ per decade of the trends are generated by vegetation degradation and expansion, respectively. This demonstrates that vegetation change would mitigate the wetting trend, particularly of the vegetation expansion. Effects of vegetation degradation in the wetting regions mainly focus on lengthening the dry season and increasing the streamflow, which subsequently decreases the rainfall infiltration^27^.

Based on the separating soil moisture trends above, I subsequently quantify the contributions of climate and vegetation change (see the Methods section). Figure 2 shows the individual contributions at global scale. In the drying zone, −0.77% and 1.99% of the decreasing soil moisture are originated from vegetation degradation and expansion, respectively. The contribution of climate change is therefore 98.78%. In the wetting zone, the contributions are −0.46% and −14.18% for the vegetation degradation and expansion, respectively, with climate change contributing 114.64% to the soil moisture increase. It demonstrates that vegetation change exhibits a weak contribution to global drying and wetting trends. The reason is mainly attributed to their small area. Climate change occurs over the entire Earth, while vegetation change only occurs in a limited region on land (only 40% of the land). Therefore, the small area of vegetation change weakens its impact on the global moisture trend.

Figure 3 further presents the contributions in the regions of soil moisture change. In the drying zone, the climate and vegetation change account for 110.87% and −10.87%, respectively, of the soil moisture decrease in the vegetation degradation regions. These values are 91.79% and 8.21% in the vegetation expansion regions. In the wetting zone, the contributions are 105.65% and −5.65% in the vegetation degradation regions and 140.21% and −40.21% in the expansion regions. Climate change still dominates the soil moisture trend. However, the regional contributions of vegetation change are much greater than those at a global scale, particularly in the vegetation degradation regions of the wetting zone. These results are very helpful for providing insight into regional land cover and water management. For example, reforestation could effectively eliminate flooding in humid regions, while deforestation could hold more water in the soil and protect against drought in arid regions. The appropriate forest planning strategy is helpful for effective management of water resources and climatic adaptation.

The spatial pattern of local contribution is also evaluated and mapped for vegetation change. The averaged soil moisture trend in the non-vegetation/change pixels in a 1.25° × 1.25° window is taken as the local contribution of climate change. Then the contributions of vegetation change are extracted in the vegetation expansion and degradation pixels. The size of the window is designed for capturing the local climate-soil moisture interaction^28^,^29^. Figure 4 further shows the contribution of vegetation change at the local sites. Vegetation change has a significantly positive contribution in South America, a negative contribution in northern Africa. The contribution is strongly correlated to soil moisture and vegetation trends. Soil moisture is low (0.217 cm^3^/cm^3^) and decreased slightly (−0.004 cm^3^/cm^3^ per decade) in the areas with a negative contribution. However, in the areas with a positive contribution, soil moisture is significantly higher (0.227 cm^3^/cm^3^) and decreases rapidly (−0.009 cm^3^/cm^3^ per decade). In addition, vegetation is relatively sparse (NDVI = 0.383, with trend of 0.007 per decade) for the negative contributions and dense (NDVI = 0.427, with trend of 0.008 per decade) for the positive contributions. These results suggest that vegetation expansion would accelerate the soil drying trend in the dry and sparsely vegetated areas. However, in the wet and dense vegetated areas, the vegetation expansion would mitigate the drying trend. The reason for this difference is due to the higher transpiration rates in the vegetated regions, which aggravates the water deficit in dry regions. In wet regions, the vegetation would store more water from rainfall, which could compensate the consumption of water from evapotranspiration^30^,^31^,^32^.

Overall, this study uses the satellite-retrieved data to separate the individual contributions of climate and vegetation changes to the soil moisture trend. The surface characteristics, particularly of land cover, strongly affect the reliability of satellite soil moisture. The validation shows that CCI soil moisture is highly consistent with the ground observation data over different land covers, with the correlation coefficient (R) and root mean square error (RMSE) range from 0.46 ± 0.23, and 0.03 to 0.09 cm^3^•cm^−3 ^33. It demonstrates that the CCI data is suitable for capturing the trend of global soil moisture. Results show that vegetation has a weak global impact due to the relatively small size of the changing areas. Regionally, the contribution of vegetation change cannot be neglected. Locally, vegetation negatively affected soil moisture variation in the dry and sparsely vegetated areas and positively in the wet and densely vegetated areas. In conclusion, climate change dominated the global soil moisture trend in both drying and wetting zones. Vegetation change served as a regulator to accelerate or decelerate the trend under the changing climate. Results of this study could support water adaptation and management at global or regional scales. As vegetation change plays limited influence on global soil moisture trend, the adaptation of global warming should be paid more attention. On the other hand, vegetation significantly influences regional soil moisture changes, and the regulation of land cover needs attention to promote regional water management (i.e., drought and flood mitigation). For example, previous studies revealed that forest have a strong ability to hold soil water^19^,^34^. Therefore, reforestation would be an effective way to mitigate the local soil drought, with its effect should be enhanced for water resource management in a changing climate.

## Methods

### Global soil moisture data set

The Climate Change Initiative (CCI) data set of the European Space Agency (ESA) is adopted for monitoring the global soil moisture trend. This data set contains fusion data with a combination of active and passive microwave satellite observations. The active data sets are generated by the University of Vienna and based on the observations from C-band scatterometer on board the European Remote Sensing (ERS) satellites (ERS-1 and ERS-2) and the Meteorological Operational Satellite (MetOp-A). The passive data sets are generated by VU University Amsterdam in collaboration with NASA based on the passive microwave observations from the Scanning Multichannel Microwave Radiometer (SMMR), Special Sensor Microwave/Imager (SSM/I), Tropical Rainfall Measuring Mission microwave imager (TRMM TMI) and the Advanced Microwave Scanning Radiometer - Earth Observing System (AMSR-E). The data set is obtained from the ESA website (http://www.esa-soilmoisture-cci.org). The accuracy is acceptable when validated by global ground-based observations, with a mean correlation coefficient (R) and root mean square error (RMSE) of 0.46 and 0.04 cm^3^/cm^3^, respectively^33^. The spatial gaps are filled by interpolation with the time series trends.

### Global NDVI data

For the vegetation change evaluation, the Global Inventory Monitoring and Modeling System (GIMMS) NDVI3g (third generation GIMMS NDVI from AVHRR sensors) is obtained from (http://ecocast.arc.nasa.gov/data/pub/gimms/3g.v0/). The data ranges from July 1981 to Dec. 2013 with a spatial resolution of 0.833 degrees. The data set subsequently is resampled to 0.25 degrees to compare with the data set of CCI soil moisture.

### Calculation of the individual contributions

A trajectory method is used to quantify the contributions of climate and vegetation change on the terrestrial water trends^19^. Soil moisture in non-change vegetation regions represents a synthetic result of climate influences and serves as a reference for isolating soil moisture alterations due to vegetation in other regions. Globally, the contributions are calculated with area weight summarization:


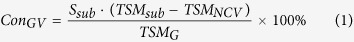



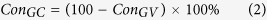


where *Con*_*GC*_ and *Con*_*GV*_ are the global contributions of climate and vegetation, *S*_*sub*_ is the area ratio of each vegetation change region in the drying or wetting areas, *TSM*_*NCV*_ and *TSM*_*sub*_ are the soil moisture trends in the non-change and change vegetation regions and *TSM*_*G*_ is the trend in the global drying or wetting zones.

At the regional and local scales, climate change and vegetation change influence soil moisture at certain sites. Their contributions are calculated as


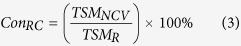



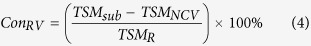


where *Con*_*RC*_ and *Con*_*RV*_ are the regional or local contributions of climate and vegetation change, *TSM*_*NCV*_ and *TSM*_*sub*_ are defined in Eqs (1) and (2) and *TSM*_*R*_ is the regional or local trend in the vegetation change regions.

## Additional Information

**How to cite this article**: Huihui, F. Individual contributions of climate and vegetation change to soil moisture trends across multiple spatial scales. *Sci. Rep.* **6**, 32782; doi: 10.1038/srep32782 (2016).

## Figures and Tables

**Figure 1:**
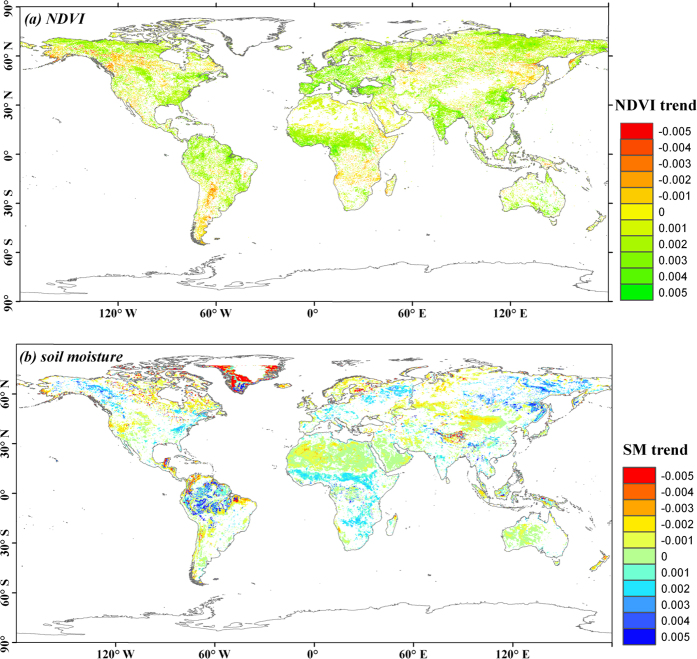
Temporal trends of global (**a**) NDVI and (**b**) soil moisture during 1982 to 2013. Only significant at p < 0.05 are presented. The non-vegetation regions are defined as the areas with a yearly average NDVI < 0.1^24^ and are removed from the analysis. All figures were generated using ArcGIS, [10.0], (URL: http://www.esri.com/), and the coordinate system is the World Geodetic System 1984 (WGS84).

**Figure 2:**
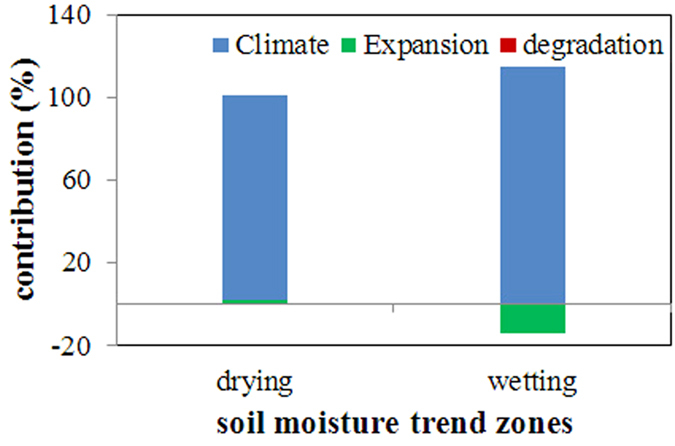
Individual contributions in the global drying and wetting zones. The summarization of the individual contributions equals 100%. The figure was generated using Microsoft Excel, [2010], (URL: https://www.office.com/).

**Figure 3:**
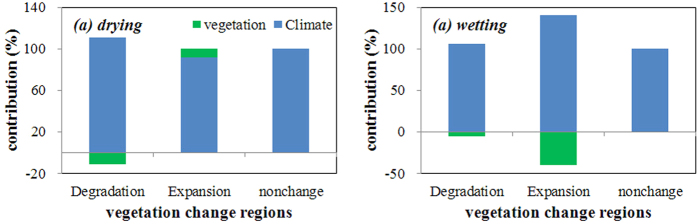
Individual contributions in the vegetation change regions of the drying and wetting zones. The summarization of the individual contributions equalled 100% in each vegetation change region (degradation, expansion and non-change). Because there is non-vegetation or change, the soil moisture trend in the non-vegetation/change regions is fully dominated by climate change. The non-vegetation regions are defined as the areas with a yearly average NDVI < 0.1^24^. The figure was generated using Microsoft Excel, [2010], (URL: https://www.office.com/).

**Figure 4:**
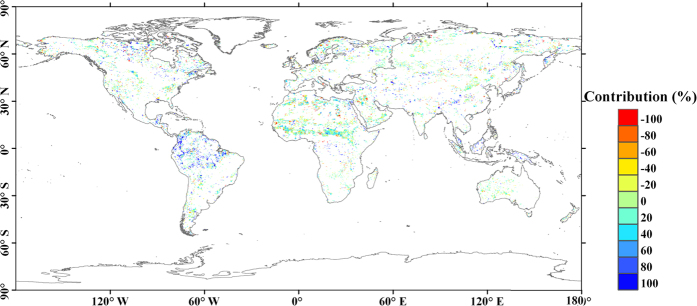
Local contribution of vegetation change. The contribution of vegetation change is calculated as *Con*_*C*_ = (100 − *Con*_*L*_) × 100%. Only trends significant at *p* < 0.05 for both soil moisture and vegetation are presented. The figure was generated using ArcGIS, [10.0], (URL: http://www.esri.com/), and the coordinate system is the World Geodetic System 1984 (WGS84).
